# Elucidating the Effects of Selenium Enrichment on the Structure and Antioxidant Properties of Selenium-Containing Proteins in Yeast Cells

**DOI:** 10.3390/antiox15030370

**Published:** 2026-03-15

**Authors:** Lixia He, Xu Wang, Jiangrong Xiao, Jie Qiao, Ying Ma, Yi He

**Affiliations:** 1 School of Modern Industry for Selenium Science and Engineering, Wuhan Polytechnic University, Wuhan 430023, China; 2 National R&D Center for Se-Rich Agricultural Products Processing, Hubei Engineering Research Center for Deep Processing of Green Se-Rich Agricultural Products, Wuhan Polytechnic University, Wuhan 430023, China; 3 School of Chemical and Environmental Engineering, Wuhan Polytechnic University, Wuhan 430023, China; 4 School of Life Science and Technology, Wuhan Polytechnic University, Wuhan 430023, China

**Keywords:** selenium biotransformation, yeast, selenomethionine, protein structure, antioxidant activity

## Abstract

Selenium (Se) enrichment in yeast represents a promising strategy for producing organic Se with high bioavailability. However, a systematic understanding of how Se incorporation alters intact protein structure and function across diverse strains remains lacking. This study investigated four yeast species (*Saccharomyces cerevisiae*, *Kluyveromyces marxianus*, *Kluyveromyces lactis*, and *Torulaspora delbrueckii*) using multi-spectroscopic and radical scavenging assays. Despite moderate growth inhibition (10.4–27.7%), all strains accumulated substantial Se (1164–2858 µg/g). Structural analysis revealed that Se induced strain-dependent protein conformational perturbations. Specifically, in selenium-enriched *Saccharomyces cerevisiae*, where Se was predominantly incorporated as selenomethionine (SeMet, 85.80%), a significant structural relaxation occurred. This was characterized by decreased rigid β-sheet content, increased flexible random coils, and a substantial enhancement in surface hydrophobicity. Crucially, Pearson correlation analysis revealed that functional enhancements were synergistically governed by specific Se speciation and secondary structural remodeling. Enhanced DPPH^•^ scavenging activity was positively correlated with changes in β-sheet and random coil structures. Selenomethionine content was also significantly correlated with increased scavenging of ^•^OH and ABTS^•+^. Consequently, *Saccharomyces cerevisiae* uniquely achieved highly significant (*p* < 0.001) antioxidant improvements, whereas other strains showed moderate or non-significant responses despite high Se yields. Our findings demonstrate that the antioxidant efficacy of selenoproteins is not solely determined by total Se content but is fundamentally driven by the targeted bioconversion of SeMet and its associated structural relaxation.

## 1. Introduction

Selenium (Se), an essential trace element, plays irreplaceable roles in redox homeostasis, immune regulation, and disease prevention across biological systems [1]. To maintain these physiological functions, adequate daily intake is essential. The recommended dietary allowance (RDA) for Se varies by country and organization, generally ranging from 55 to 75 μg per day for adults, with specific values differing by sex and the biomarker used to establish requirements [2,3,4,5]. To meet these physiological demands, humans primarily rely on their diet. Natural foods recognized as rich sources of Se include seafood, meats, poultry, eggs, and cereals [5]. Among plant-based sources, Brazil nuts are the most concentrated dietary source, though their Se content is highly variable [6]. Crucially, the Se concentration in these natural foods depends heavily on regional soil conditions, which often leads to fluctuating intake and geographical deficiencies. Its deficiency is associated with pathologies such as Keshan disease, whereas optimal intake enhances antioxidant defenses and reduces the risk of cancer [7]. The development of effective and safe Se supplements is, therefore, a key objective in nutritional science and functional food innovation. Importantly, the bioavailability and safety of different forms of Se dictate their efficacy. Compared to inorganic counterparts, organic Se compounds—where Se is covalently bonded to carbon—demonstrate superior absorption and lower acute toxicity. The primary sources of organic Se are selenoamino acids, notably selenomethionine (SeMet) and selenocysteine (SeCys), which are the major forms in plants and animal tissues, respectively [2]. Other highly bioactive organic sources include Se-methylselenocysteine (MeSeCys) found in Se-enriched yeast and allium vegetables, selenoneine from marine sources, and recently identified selenosugars [8]. These advantages establish organic Se as the gold standard for nutraceuticals [9,10].

The safe and efficient conversion of inorganic Se into organic Se is the key path to develop Se rich products. Among the three biotransformation strategies of plant transformation, animal transformation, and microbial transformation, microbial enrichment is becoming the core technology of the emerging Se industry [11,12]. It uses inorganic Se (such as selenite) as a raw material to produce organic Se with high nutrition and low toxicity, which can effectively meet the health needs of Se-deficient people. Compared with the limitations of low enrichment efficiency and long cycle of plant transformation, microbial transformation stands out with its advantages of strong metabolic activity and low culture cost [13]. Among them, yeast is a carrier with a strong ability to transform inorganic Se, and it is also a safe and practical microorganism [14]. Therefore, enriching yeast with Se presents an attractive strategy for developing functional food ingredients and nutraceuticals, aiming to enhance Se bioavailability and antioxidant efficacy beyond what is achievable with inorganic supplements.

Yeast, as a single-cell eukaryotic model organism that has been used for thousands of years, has become an important carrier of modern biological research with the advantages of simple culture and a short cycle [15]. The bioconversion ability of inorganic Se is particularly outstanding; by adding inorganic Se to the culture medium, yeast can independently transform it into organic forms such as selenoprotein and selenoamino acids [16,17,18,19]. The effect of biologically bound Se in yeast is significantly higher than that of inorganic Se compounds in terms of direct scavenging of oxygen-free radicals and inhibiting lipid peroxidation [20]. Yeast accelerates Se uptake by upregulating the expression of Se transporters and integrates SeMet and SeCys into the protein skeleton as the main forms [21]. It is worth noting that the incorporation of Se changes the structural basis of protein [21]. When Se atom replaces the sulfur atom in methionine, its larger atomic radius and electronegativity difference induce secondary bond breakage and hydrophobic group rearrangement, and change the spatial conformation of protein [22,23].

To contextualize these structural shifts, one must consider yeast’s native redox architecture. Endogenous reactive oxygen species (ROS) trigger robust biochemical attenuation pathways driven by enzymatic (e.g., superoxide dismutase, catalase) and non-enzymatic (e.g., glutathione (GSH), nicotinamide adenine dinucleotide phosphate (NADPH)) scavengers [24,25]. At the molecular level, this defense is genetically orchestrated by oxidant-sensitive transcription factors, predominantly the Yap1 and Skn7 signaling pathways [26]. Crucially, Se biotransformation amplifies these native systems. The incorporation of selenomethionine establishes a highly efficient, self-regenerating catalytic cycle via the GSH network [27]. Thus, selenium-induced protein structural reorganization operates synergistically with these genetic and biochemical pathways to fundamentally enhance cellular antioxidant capacity.

At present, it has been found that after Se-enriched culture of *Saccharomyces cerevisiae* (*S*. *cerevisiae*), the infrared spectrum confirmed that Se was embedded in Se-O bond and C-Se-O bond, causing characteristic peak shift and hydroxyl polarization [28]. Structural changes directly drive the functional jump. The scavenging ability of Se-rich proteins on 1,1-diphenyl-2-picrylhydrazyl (DPPH^•^), hydroxyl (^•^OH) and other free radicals is much higher than that of ordinary proteins [28]. Some studies on Se-rich peptides obtained from enzymatic hydrolysis of Se-rich proteins also confirmed the role of Se integration in improving the antioxidant capacity of biomolecules [20]. In addition, Se can activate the antioxidant enzyme system of yeast (*S*. *cerevisiae* or *Candida utilis*) and induce post-translational modification, which can synergistically enhance the overall antioxidant defense of cells [29,30].

While *S*. *cerevisiae* is the primary workhorse in traditional baking and alcoholic fermentations due to its exceptional stress tolerance, the industry increasingly relies on non-Saccharomyces species to diversify product profiles. Specifically in the context of fruit processing and fermentation, these selected strains play indispensable roles. *S. cerevisiae* remains the fundamental driver for traditional fruit alcoholic beverages, most notably grape wines and apple ciders. The thermotolerant, food-grade species *Kluyveromyces marxianus* (*K*. *marxianus*) and *Kluyveromyces lactis* (*K. lactis*) are highly valued in dairy fermentations for their unique lactose-utilizing capabilities [31]. Additionally, *Torulaspora delbrueckii* (*T. delbrueckii*) is widely commercialized in modern winemaking and sourdough production to enhance sensory complexity and aromatic notes [32]. Given their distinct metabolic characteristics and established safety, exploring this diverse spectrum of yeasts for Se enrichment presents a promising avenue for developing tailored functional foods. Despite the documented benefits of selenium-enriched yeast, a critical research gap remains: a systematic understanding of how Se incorporation alters the intact protein structure across diverse yeast species and whether these conformational changes directly dictate the enhancement of antioxidant efficacy. Previous studies have largely relied on indirect methods or single strains, lacking a comprehensive, multi-strain comparison at the intact protein level. Furthermore, current practices often evaluate selenium-enriched yeast based solely on total Se accumulation, assuming higher content naturally dictates stronger bioactivity. The core advantage of our proposed approach is shifting this focus to protein structural reorganization.

To bridge this gap, this study was designed with a systematic workflow: First, we evaluated and compared the Se enrichment efficiency and biomass response across four distinct yeast species (*S*. *cerevisiae*, *K*. *marxianus*, *K*. *lactis*, and *T*. *delbrueckii*). Second, we extracted the intact selenium-containing proteins and elucidated their strain-specific structural modifications and Se speciation. Finally, we assessed the intrinsic free radical scavenging activity of these intact proteins. By integrating these steps, we aimed to establish a direct structure–function relationship that can guide the precision selection and design of yeast strains for tailored selenium-enriched functional ingredients.

## 2. Materials and Methods

### 2.1. Materials

Sodium selenite, nitric acid, glucose and agar powder were obtained from Guoyao Group Chemical Reagent Co., Ltd. (Shanghai, China). Yeast powder and tryptone were purchased from Biosharp Life Sciences Co., Ltd. (Hefei, China). The BCA protein detection kit was supplied by Beyotime Biotechnology Co., Ltd. (Shanghai, China). Additionally, DPPH^•^, 2,2′-azino-bis(3-ethylbenzothiazoline-6-sulfonic acid) (ABTS^•+^), salicylic acid, and ferrous sulfate were sourced from McLean Technology Co., Ltd. (Shanghai, China). All chemicals used in this study were of analytical grade.

### 2.2. Microorganism and Cultivation

The four yeast strains utilized in this study were *Saccharomyces cerevisiae* (*S*. *cerevisiae*) CICC 1575, *Kluyveromyces lactis* (*K*. *lactis*) CICC 1572, *Kluyveromyces marxianus* (*K*. *marxianus*) CICC 31189, all obtained from the China Industrial Microbial Strain Preservation and Management Centre, along with *Torulaspora delbrueckii* (*T*. *delbrueckii*) G-1, which was isolated from Guankou grapes collected in Enshi, Hubei Province, China, and its taxonomic identity was authenticated through standard morphological evaluation and 26S rDNA-based molecular phylogenetic analysis.

The Yeast Extract Peptone Dextrose (YPD) liquid medium was prepared in the laboratory with the following mass concentrations: peptone at 20 g/L, dextrose at 20 g/L, and yeast extract at 10 g/L. The independent variable was the presence of Se in the culture medium. Each strain was cultivated under two conditions: an experimental group grown in selenium-enriched YPD liquid medium (supplemented with 20 μg/mL sodium selenite) and a control group cultured in standard selenium-free YPD medium. This specific concentration of sodium selenite was selected based on prior studies indicating it optimally balances yeast cell viability with the efficient biosynthesis of SeMet [33]. For clarity and brevity in data presentation, the control samples cultivated in standard medium are designated with specific group IDs: Sac (derived from *S*. *cerevisiae*), Kll (derived from *K*. *lactis*), Klm (derived from *K*. *marxianus*), and G-1 (derived from *T*. *delbrueckii*). Correspondingly, the experimental samples cultivated in the selenium-enriched medium are referred to as Se-Sac, Se-Kll, Se-Klm, and Se-G-1, respectively. Briefly, a seed culture with an optical density (OD600) of 0.8 was inoculated at a 5% (*v*/*v*) ratio into 100 mL of YPD liquid medium within a 250 mL Erlenmeyer flask. The cultivation time was specified as 36 h [34].

Dependent variables comprised endpoint biomass yield (measured as dry cell weight), Se accumulation, protein structural characteristics, and antioxidant activity. For analysis, yeast cells were harvested by centrifugation at 7155× *g* for 10 min using a high-speed refrigerated centrifuge (Neo 15R; LiShen Scientific Instrument Co., Ltd., Shanghai, China) [35]. The pellets were washed three times with deionized water, which was obtained from a laboratory water purification system (CL-40W; Pinguang Instrument Equipment Co., Ltd., Wuhan, China). Subsequently, the samples were freeze-dried using a freeze dryer (LGJ-12F; Beijing Sihuan Foring Technology Development Co., Ltd., Beijing, China) at −70 °C and <10 Pa for 72 h to obtain both selenium-enriched and control dry yeast powder. All cultivations and subsequent analyses were performed using three independent biological replicates.

### 2.3. Preparation of Yeast Proteins

Protein extraction was conducted based on previous research, with modifications [36]. First, 0.1 g of dry yeast powder was weighed and placed into a test tube. Subsequently, 10 mL of ultrapure water was added, and the cells were sonicated using an ultrasonic processor (300 W, 10 s on/5 s off) for 15 min in an ice bath to prevent thermal denaturation. The proteins were precipitated using cold acetone at a 1:1 (*v*/*v*) ratio overnight at −20 °C, followed by centrifugation of the precipitate at 7155× *g* for 10 min. This precipitation step also effectively separated the intact proteins from any unincorporated free inorganic or organic Se remaining in the supernatant. Finally, the pellet was reconstituted in Phosphate-Buffered Saline (PBS) buffer (pH 7.4). To ensure the complete removal of any residual free Se and small-molecule metabolites, the protein solution was dialyzed using a dialysis membrane with a molecular weight cut-off (MWCO) of 3 kDa against distilled water at 4 °C to obtain the yeast protein solution, which was then freeze-dried to yield protein dry powder. It should be noted that this extraction protocol yields a total soluble intracellular protein fraction. This approach was deliberately chosen to evaluate the collective structural and functional modifications of the comprehensive yeast proteome following Se enrichment, rather than isolating a single specific protein.

### 2.4. Determination of Biomass and Se Content in Yeast

The freeze-dried selenium-enriched yeast and standard yeast powders were accurately weighed to determine the biomass and total Se content. According to a previously reported method, the procedure was modified to utilize ultrasound-assisted acid digestion [34]. Prior to digestion at 170 °C, 0.100 g of the sample was weighed and treated with 6 mL of HNO_3_. After digestion, a 2% nitric acid solution was added to adjust the final volume to 10 mL. The blank was prepared under the exact same digestion conditions and analyzed simultaneously to monitor and subtract background contamination. The analysis was performed using an Agilent 7900 inductively coupled plasma mass spectrometry instrument (ICP-MS, Agilent Technologies, Beijing, China). The absolute quantification of total selenium was strictly performed utilizing an external standard calibration method, which demonstrated excellent linearity (R^2^ > 0.999). The ^78^Se isotope was monitored, and potential argon-based polyatomic interferences (e.g., ^40^Ar^38^Ar^+^) were effectively eliminated by operating the collision reaction cell in He mode with kinetic energy discrimination. The instrumental parameters were as follows: radio frequency (RF) power, 1550 W; carrier gas, argon; collision gas, helium; atomizer chamber temperature, 2 °C; plasma, auxiliary, carrier, and compensation flow rates, 15, 1, 1, and 1 L/min, respectively; sample uptake delay, 15 s; instrumental stabilization delay, 8 s; replicates, 3; and replicate read time, 0.3 s. A platinum cone was used for both the sampling and extraction cones.

### 2.5. Determination of Protein Content and Protein Se Content

Protein concentration was determined using the bicinchoninic acid (BCA) assay with a commercial BCA protein assay kit (Beyotime Biotechnology, Shanghai, China). To ascertain the protein-bound Se content, approximately 1 mL of the reconstituted protein sample (1 mg/mL) was transferred into a 50 mL graduated tube and subjected to microwave digestion. The microwave digestion procedure was identical to the method described in Section 2.4. The protein-bound Se content was assessed using the Agilent 7900 ICP-MS system and normalized per gram of protein.

### 2.6. Se Speciation Analysis

Protein Se speciation was determined according to a previously reported method with modifications [37]. A 1 mL aliquot of the sample was mixed with an enzymatic solution containing Pronase E (0.4 mg/mL, 7 units/mg) and proteinase K (0.4 mg/mL, 30 units/mg) at an enzyme-to-substrate ratio of 1:20 (*w*/*w*). After 2 h of ultrasonication and overnight incubation at 37 °C, the mixture was centrifuged at 7155× *g* for 10 min. The supernatant was collected and filtered through a 0.45 μm membrane. Standard curves were generated using authentic standards of SeMet, selenocystine (SeCys_2_), MeSeCys, Se(IV), and Se(VI). The determination of Se speciation forms was carried out using high-performance liquid chromatography combined with inductively coupled plasma tandem mass spectrometry (HPLC-ICP-MS/MS, Agilent Technologies, Beijing, China). The separation of Se compounds was performed on a Hamilton PRP-X100 anion exchange chromatography column (4.1 mm × 250 mm, 10 μm), maintained at 30 °C in a constant temperature chamber. The mobile phase consisted of 10 mM citric acid (pH 5.0) supplemented with 2% methanol to enhance the signal. The injection volume was set to 10 μL, the flow rate was 1 mL/min, and the detection time was 9 min. Under these chromatographic conditions, baseline resolution (Rs > 1.5) was achieved for all targeted Se species.

The specific retention times for the targeted Se species were 2.28 min for SeCys2, 2.99 min for MeSeCys, 3.54 min for Se(IV), 5.32 min for SeMet, 8.22 min for Se(VI). Regarding the specific operational parameters of the ICP-MS/MS system, the radio frequency (RF) power was set at 1550 W. To effectively eliminate polyatomic interferences, the collision/reaction cell was operated using He gas. Furthermore, the key energy and voltage parameters for ion transmission were configured with an extract lens voltage of −5.0 V and an octopole bias of −100.0 V.

### 2.7. UV-Vis Absorption Spectroscopy

UV-Vis absorption spectra were analyzed using a UV–visible spectrophotometer (Thermo, Waltham, MA, USA). Solutions of ordinary yeast protein and selenium-enriched yeast protein were prepared at a concentration of 0.1 mg/mL. Measurements were performed in a 1 cm path-length quartz cuvette using PBS as the blank, with appropriate baseline correction. Spectral scanning was conducted over the wavelength range of 200 to 800 nm.

### 2.8. Fourier-Transform Infrared Spectroscopy (FTIR)

For FTIR analysis, 1 mg of the freeze-dried ordinary yeast protein powder or selenium-enriched yeast protein powder was mixed with potassium bromide (KBr) at a ratio of approximately 1:100 (*w*/*w*). The mixture was ground evenly, placed into a mold, and pressed into transparent pellets using a hydraulic press. The resulting samples were subsequently analyzed using an FT-IR spectrometer (Perkin Elmer, Waltham, MA, USA), and the spectra were recorded over the wavenumber range of 400 to 4000 cm^−1^.

### 2.9. Fluorescence Spectroscopy

To investigate the intrinsic protein fluorescence properties of the samples, 0.1 mg/mL solutions of ordinary yeast protein and selenium-enriched yeast protein were prepared. The excitation wavelength was set to 252 nm using a Hitachi F-4600 fluorescence spectrophotometer (Hitachi Co., Tokyo, Japan), and the emission spectra were recorded over the wavelength range of 200 to 800 nm.

### 2.10. Circular Dichroism Spectroscopy

The circular dichroism (CD) spectroscopy of selenium-enriched yeast protein and regular yeast protein was observed on Jasco J-1500 circular dichroism spectrometer (Tokyo, Japan). The protein samples were dissolved in a Phosphate Buffer (PB, pH 7.0) to achieve a final concentration of 0.1 mg/mL. The prepared solutions were then added to quartz cells with a path length of 1 cm. The corresponding PBS (pH 7.4) was used as the blank reference, and rigorous baseline correction was performed by subtracting the blank spectrum prior to sample analysis. The CD spectra were scanned at 250–195 nm at a scanning speed of 50 nm/min [38]. The relative proportions of secondary structural elements (α-helix, β-sheet, β-turn, and random coil) were estimated by deconvoluting the spectra using the instrument’s built-in “Protein Secondary Structure Estimation” software (Spectra Manager, Version 2.02.01), applying Yang’s reference model.

### 2.11. Determination of Surface Hydrophobicity (H_0_)

The surface hydrophobicity of both the control and selenium-enriched yeast protein solutions was determined utilizing the 8-anilino-1-naphthalenesulfonic acid (ANS) fluorescence probe method. Protein samples were diluted in PBS to concentrations of 0.25, 0.50, 0.75, and 1.0 mg/mL. Following this, 4 mL of each diluted protein sample was mixed with 20 μL of an 8 mM ANS solution and incubated in the dark for 10 min. After subtracting the background fluorescence of the ANS blank, the fluorescence intensity was measured. The determination was conducted under the following conditions: excitation at 390 nm, emission detection at 470 nm, and a slit width of 5 nm. A standard curve was generated by plotting protein concentration (mg/mL) against the corresponding fluorescence intensity. A linear regression equation was derived (R^2^ > 0.99), with the calculated slope representing the surface hydrophobicity index (H_0_) of the protein sample.

### 2.12. Detection of Antioxidant Activity

The scavenging capacities of the protein samples against ABTS^•+^, DPPH^•^, and ^•^OH radicals were evaluated based on previously reported methods [39,40], with minor modifications.

The scavenging capacity against ABTS^•+^ radicals was evaluated using a modified method. Briefly, an ABTS stock solution (7.4 mmol/L) was mixed with a 2.6 mmol/L potassium persulfate solution in equal volumes and incubated in the dark at 4 °C for 16 h to ensure complete and stable generation of the ABTS^•+^ radical cation. Prior to use, the working solution was diluted with PBS to achieve an absorbance of 1.0 ± 0.2 at 734 nm. For the assay, 1 mL of the yeast protein solution (1 mg/mL) was mixed with 4 mL of the working solution. After a 30 min incubation in the dark at 37 °C, the absorbance was measured at 734 nm. The results were expressed as a radical scavenging percentage, which facilitates a direct relative comparison of antioxidant capacity between the native and structurally reorganized selenium-enriched proteins at a standardized concentration.

For the DPPH^•^ radical scavenging assay, the procedure was adapted from previously established protocols. Specifically, 1 mL of the protein solution (1 mg/mL) (which was previously filtered through a 0.45 μm membrane to ensure optical clarity) was mixed with an equal volume of a 0.1 mg/mL DPPH solution dissolved in methanol, bringing the final reaction volume to 2 mL. The mixture was shaken well and allowed to react in the dark for 30 min. Subsequently, the absorbance of the reaction mixture was measured at 517 nm.

The ^•^OH scavenging activity was determined utilizing the salicylic acid method. First, 1 mL of the sample solution (1 mg/mL) was placed in a test tube, followed by the addition of a 9 mmol/L salicylic acid solution and a 9 mmol/L ferrous sulfate solution. The reaction was then initiated by introducing H_2_O_2_ (8.8 mmol/L). The mixture was shaken thoroughly, and the absorbance was recorded at 510 nm.

To eliminate redundancy, the radical scavenging rates (%) for all three aforementioned assays were uniformly calculated using the following consolidated equation:

(1) Radical scavenging rate (%) = [1 − (A_i_ − A_j_)/A_0_] × 100

In this formula, A_0_ is the absorbance of the blank control, A_i_ is the absorbance of the reaction mixture, and A_j_ is the absorbance of the sample background.

### 2.13. Statistical Analysis

The measured values were expressed as the mean ± standard deviation, derived from at least three parallel replicates. Data analysis was conducted using SPSS statistical software (Version 27.0), with a significance level set at *p* < 0.05. Prior to parametric testing, the normality of the data distribution and the homogeneity of variances were confirmed. To assess the effect of Se treatment on growth and protein production in each strain, paired *t*-tests were employed (e.g., Se-Sac vs. Sac). To compare the Se accumulation capacity across all strains, a one-way ANOVA was conducted, followed by Fisher’s Least Significant Difference (LSD) post hoc test for multiple comparisons.

A Pearson correlation analysis was performed using SPSS software. Specifically, this analysis evaluated the associations among the relative percentages of SeMet, the changes in protein secondary structural contents, and the enhancements in antioxidant activities across different yeast strains. A heatmap was generated to visualize the correlation coefficients (r) and statistical significance (*p*-values).

## 3. Results

### 3.1. Strain-Specific Biomass Response and Se Accumulation

In industrial production, selenium-enriched yeast is typically cultivated in media supplemented with 20 to 30 mg/L of Se(IV) [41]. Among these concentrations, 20 mg/L of Se(IV) optimally promotes the biosynthesis of SeMet and SeCys [33]. Therefore, we cultured *S*. *cerevisiae*, *K*. *lactis*, *K*. *marxianus* and *T*. *delbrueckii* at the Se(IV) treatment of 20 mg/L.

All strains exhibited growth inhibition under Se stress (Table 1), with Sac and Klm showing marked reductions of 10.40% and 27.70%, respectively. In contrast, Kll and G-1 demonstrated greater tolerance, with only marginal declines of 1.82% and 7.69%. Despite this growth suppression, substantial Se bioaccumulation occurred across all strains, with total Se content ranging from 1164.00 to 2858.70 μg/g dry weight (Table 1). These enrichment levels are highly competitive and frequently exceed typical commercial selenium-enriched yeast products, which generally contain 1000 to 2000 μg/g of Se. Although certain adapted strains or specific species like *Rhodotorula glutinis* can achieve hyper-accumulation levels exceeding 7000 μg/g under optimized conditions, such extreme accumulation often severely reduces biomass yield and compromises the efficiency of organic bioconversion [42]. Therefore, the accumulation levels observed in our study represent an optimal physiological balance. They achieve commercially viable Se concentrations while facilitating high-quality organic biotransformation. Notably, bioaccumulation efficiency varied dramatically: Kll achieved exceptional accumulation (2858.70 μg/g, a 737-fold increase compared to control), while Sac also exhibited significant enrichment that surpassed Klm and G-1.

In selenium-enriched yeast, approximately 90% of Se is present in organic forms, with the majority bound to proteins [17]. Notably, over 60% of the total Se exists as SeMet [43]. The inverse relationship between biomass reduction and Se accumulation suggests underlying metabolic trade-offs. Previous studies indicate that exogenous Se induces endogenous metabolic disturbances in *S*. *cerevisiae*, particularly disrupting amino acid metabolism and depleting GSH levels [44], providing a mechanistic basis for the observed growth inhibition. The strain-specific differences in biomass and Se accumulation, as shown in Table 1, are likely attributable to inherent variations in redox metabolism and antioxidant strategies among the four yeast strains. Specifically, the consistently higher parameters observed in Se-Kll compared to Se-Sac, including greater biomass retention and Se accumulation, highlight distinct metabolic trade-offs. The severity of biomass decline, most pronounced in Klm and Sac, likely reflects their distinct metabolic responses to Se stress [45]; for instance, exogenous Se induces more severe endogenous metabolic disturbances and GSH depletion in *S*. *cerevisiae*. In contrast, strains such as Kll and G-1 may activate compensatory pathways to mitigate Se toxicity, contributing to their relative resilience [46]. To explain the superior performance of Kll across these studied parameters compared to Sac, their distinct metabolic adaptations must be considered. While Sac possesses a robust selenite uptake capacity along with an efficient GSH-dependent reduction system [44], this bioconversion comes at a higher cost to general cellular growth. Meanwhile, the highly active pentose phosphate pathway in Kll results in elevated NADPH production [47], which drives the reduction of sodium selenite and supports antioxidant defense mechanisms [48]. This robust NADPH supply allows Kll to efficiently biotransform Se while maintaining superior cellular homeostasis, thereby sustaining higher basal metabolic parameters than Sac.

### 3.2. Impact of Se on Protein Yield and Se-Containing Proteins Formation

The strain-specific responses of protein content and protein-bound Se to Se enrichment were delineated in Table 1. Across all samples, total protein constituted 45–60% of dry biomass (Table 1), consistent with established literature values [12,41,49]. Notably, under identical Se treatment, significant reductions in protein yield were observed in Se-Sac, Se-Kll, and Se-G-1 compared to their respective controls, with decreases ranging from 9.69% to 19.45%. This aligns with prior reports of selenium-induced proteotoxic stress, which can be mechanistically attributed to impaired synthesis of membrane channels and ion transport proteins under sustained Se exposure [49].

Conversely, Klm exhibited a unique adaptive response, with Se-Klm demonstrating significantly elevated protein content. This phenomenon likely stems from metabolic pathway rewiring that enhances the production of stress-mitigating proteins [12]. Critically, despite these divergent impacts on total protein yield, Se enrichment universally and dramatically enhanced Se incorporation into the protein fraction (Table 1). Protein-bound Se concentrations surged 550- to 700-fold in enriched yeasts, reaching levels of 1000–5000 μg/g, in stark contrast to control levels of merely 3–7 μg/g. This biotransformation efficiency markedly surpassed the previously documented 133-fold increase in *S*. *cerevisiae* selenoproteins, unequivocally confirming superior Se assimilation capabilities across these four yeast strains [28]. The accumulated Se exists predominantly as organic species, with SeMet and SeCys constituting the primary molecular forms [50]. This indicates efficient non-specific substitution of sulfur analogs during protein biosynthesis.

To further elucidate the specific molecular forms of the incorporated Se, we conducted a quantitative speciation analysis. As detailed in Table 2, both the absolute concentrations and the relative percentage distributions of the identified Se species were determined. It is important to note that a significant mass balance gap exists when comparing the sum of these absolute extractable Se species to the total Se content of the proteins (Table 1). This phenomenon, frequently encountered in speciation analysis of complex biological matrices, is primarily attributed to the inherent limitations of the mild enzymatic hydrolysis (Pronase E and proteinase K) required to preserve Se speciation. Unlike aggressive acid digestion, incomplete enzymatic cleavage of the highly cross-linked yeast protein matrix leaves a considerable fraction of Se bound within unhydrolyzed macro-peptides or insoluble aggregates, which are inevitably excluded prior to HPLC-ICP-MS injection.

Nevertheless, evaluating the relative distribution of this readily extractable, bio-accessible fraction reveals significant strain-dependent disparities (Table 2). In Se-Sac and Se-Kll, Se primarily existed as SeMet, accounting for 85.80% and 60.53% of the identified Se, respectively. Although both strains were subjected to identical Se stress conditions, this notable difference in SeMet concentration is fundamentally driven by their intrinsic genetic and metabolic disparities rather than the external stress itself. *S*. *cerevisiae* possesses a highly focused sulfur assimilation pathway that overwhelmingly favors the conversion of inorganic Se into SeMet. In contrast, *K*. *lactis* employs a more divergent metabolic routing; despite achieving higher total Se accumulation (Table 1), it partitions a substantial portion of the assimilated Se into SeCys_2_ (31.16%, compared to only 3.57% in Se-Sac), which consequently restricts its relative SeMet yield. Meanwhile, in stark contrast to Sac and Kll, SeCys_2_ was the predominant form overall in Se-Klm and Se-G-1, constituting 56.71% and 61.56%, respectively. Inorganic Se species [Se(IV) and Se(VI)] consistently remained at low levels across all samples. The predominance of organic Se forms, particularly the high SeMet content in Se-Sac and Se-Kll, is highly advantageous from a nutritional perspective. Compared to inorganic Se, SeMet exhibits not only higher bioavailability [51] but also influences multiple metabolic pathways, exerting positive regulatory effects such as modulating gut microbiota and enhancing intestinal barrier function [14,52].

### 3.3. Selenium-Induced Alterations in Protein Structure

Multi-technique analysis demonstrates that the incorporation of Se induces strain-dependent structural perturbations while preserving the integrity of the core protein backbone. The subsequent techniques reveal progressive insights into these alterations.

#### 3.3.1. UV-Vis Spectral Characteristics

**Figure 1:**
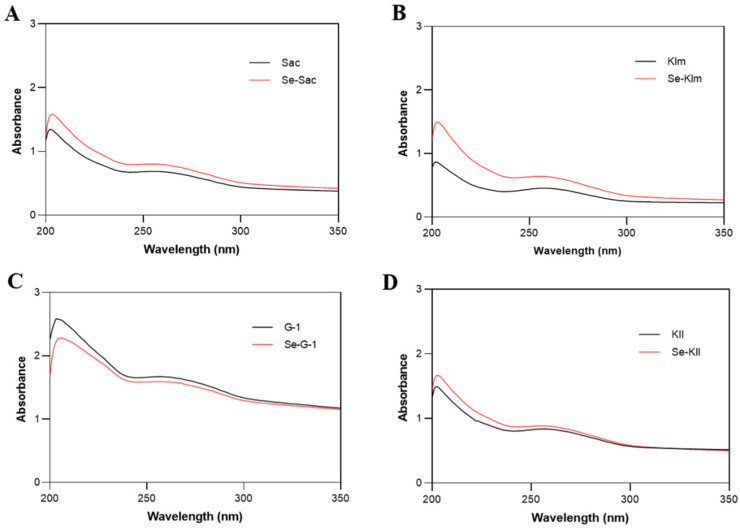
Comparative UV-Vis absorption spectra of protein extracts derived from yeast strains cultivated with and without selenium supplementation: (**A**) Sac; (**B**) Klm; (**C**) G-1; (**D**) Kll. Sac = *Saccharomyces cerevisiae*; Klm = *Kluyveromyces marxianus*; G-1 = *Torulaspora delbrueckii*; Kll = *Kluyveromyces lactis*.

UV spectral analysis of proteins from all strains revealed characteristic peaks at approximately 200 nm (n→π* transition of peptide bonds) and 250 nm (aromatic amino acid side chains) (Figure 1) [53,54]. Building on this foundation, we observed a consistent blue shift at 250 nm in selenium-binding proteins compared to the controls. As reported by Yang et al. [55], Se interaction with C=O groups in selenium-chelated wheat peptides altered electronic transitions, leading to similar spectral shifts. The increased peak intensities in Se-Sac, Se-Klm, and Se-Kll further corroborate the interactions between Se and peptide bonds, potentially through charge transfer complexes or conformational changes that expose chromophores [55,56,57].

#### 3.3.2. FTIR Spectroscopy

**Figure 2:**
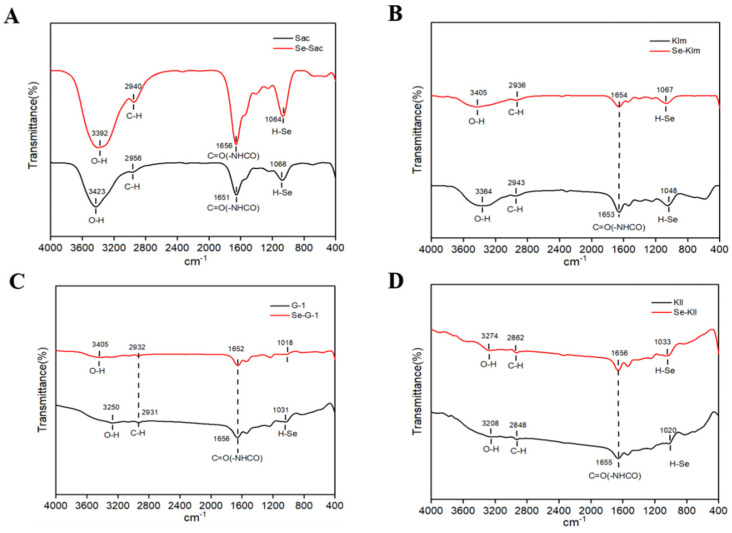
FTIR analysis of protein extracts derived from yeast strains cultivated with and without selenium supplementation: (**A**) Sac; (**B**) Klm; (**C**) G-1; (**D**) Kll. Sac = *Saccharomyces cerevisiae*; Klm = *Kluyveromyces marxianus*; G-1 = *Torulaspora delbrueckii*; Kll = *Kluyveromyces lactis*.

The chemical structure of yeast protein was investigated and confirmed using Fourier-Transform Infrared Spectroscopy (FTIR). The infrared spectral analysis revealed that both the selenium-containing protein and the control protein exhibited certain similarities, displaying characteristic absorption peaks of hydroxyl groups between 3600 cm^−1^ and 3200 cm^−1^ (Figure 2). This observation indicates the presence of intramolecular or intermolecular hydrogen bonds in both the control and selenium-containing proteins [58]. The peaks located at 2900 cm^−1^ and 1640 cm^−1^ in all samples were attributed to the stretching vibration of C-H and the bending vibration of C=O, respectively [59]. Notably, in the Se-Sac-derived proteins, redshifts were observed in the absorption peaks of C-H and O-H, while blueshifts were noted in the absorption peaks of O-H in the other three selenized yeast proteins. These findings suggest that the incorporation of Se resulted in alterations to the hydrogen bonding characteristics of the proteins [55,58].

#### 3.3.3. Fluorescence Spectral Characteristics

**Figure 3:**
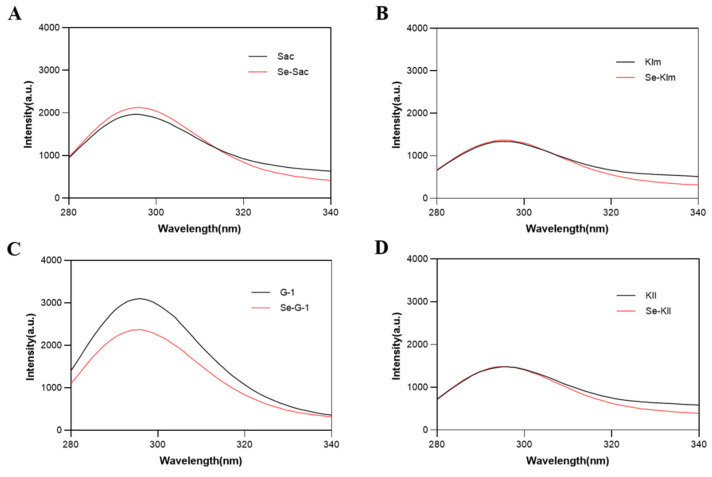
Fluorescence spectroscopy of protein extracts derived from yeast strains cultivated with and without selenium supplementation: (**A**) Sac; (**B**) Klm; (**C**) G-1; (**D**) Kll. Sac = *Saccharomyces cerevisiae*; Klm = *Kluyveromyces marxianus*; G-1 = *Torulaspora delbrueckii*; Kll = *Kluyveromyces lactis*.

The presence of tryptophan (Trp), tyrosine (Tyr), and phenylalanine (Phe) residues in protein molecules can generate fluorescence, with Trp residues being the most sensitive to changes in fluorescence [58,60]. All samples exhibited an absorption peak at approximately 300 nm (Figure 3). The intensity of the absorption peak for Se-Sac surpassed that of Sac (Figure 3A), which may stem from the binding interactions between Se and proteins, leading to the exposure of a greater number of aromatic amino acids [56]. Additionally, when compared to the control group, the maximum protein emission values (approximately 300 nm) for Se-Sac and Se-Kll demonstrated a slight blue shift, indicating a decrease in the environmental polarity surrounding the Trp residues, suggesting a more hydrophobic environment. This transformation may be induced by selenium-induced conformational changes, the exposure of tryptophan residues that are typically buried within the protein structure, or direct electron perturbation from nearby SeCys residues [39,56,60]. However, the changes observed in Klm and G-1 proteins are minimal.

#### 3.3.4. Secondary Structure (CD)

The secondary structures of the four yeast proteins were predominantly characterized by β-sheets, followed by random coils, β-turns, and α-helices (Table 3). Following Se enrichment, a universal and statistically significant structural transition occurred: the proportion of rigid β-sheets significantly decreased across all four yeast strains. Specifically, in Se-Sac, the β-sheets content dropped from 64.37% to 55.27%, accompanied by significant increases in random coils, β-turns, and α-helices (*p* < 0.05). Similarly, Se-G-1 and Se-Kll demonstrated significant reductions in β-sheets alongside marked increases in flexible random coil content (*p* < 0.05 or *p* < 0.001). Meanwhile, Se-Klm experienced a massive reduction in β-sheets (from 61.17% to 36.90%) coupled with a dramatic surge in α-helices. Overall, compared to ordinary yeast proteins, all selenium-enriched yeast proteins exhibited a pronounced loss of rigid β-sheets structures, indicating a profound shift toward more flexible or alternative conformations. To visualize these conformational shifts, representative far-UV CD spectra of the extracted proteins from all tested strains are provided in Figure S1 of the Supplementary Materials.

Luo et al. confirmed that the secondary structure of peanut protein isolate undergoes changes following Se treatment, specifically characterized by an increase in the percentages of α-helix and β-sheet, alongside a decrease in the percentage of β-turn [61]. Similarly, Zhang et al. found that, compared to ordinary brown rice protein, the percentage of β-sheet in selenium-enriched brown rice protein increased, while the percentage of α-helix decreased [59]. Collectively, these studies indicate that Se enrichment can significantly influence the secondary structure of proteins. On one hand, Se enrichment alters the composition of proteins and amino acids in yeast, thereby modifying the spatial structure of proteins [62,63]. On the other hand, methionine is randomly replaced by SeMet, which is then incorporated into yeast proteins [63,64]. Additionally, the differences in atomic size and ionization conditions between Se and sulfur contribute to the observed changes in protein structure [62,65]. Specifically, in our yeast proteins, these elemental differences create internal steric disruptions that force the rigid β-sheets cores to unwind, ultimately modifying the overall spatial conformation into a looser, more solvent-exposed architecture.

#### 3.3.5. Surface Hydrophobicity (H_0_)

**Figure 4:**
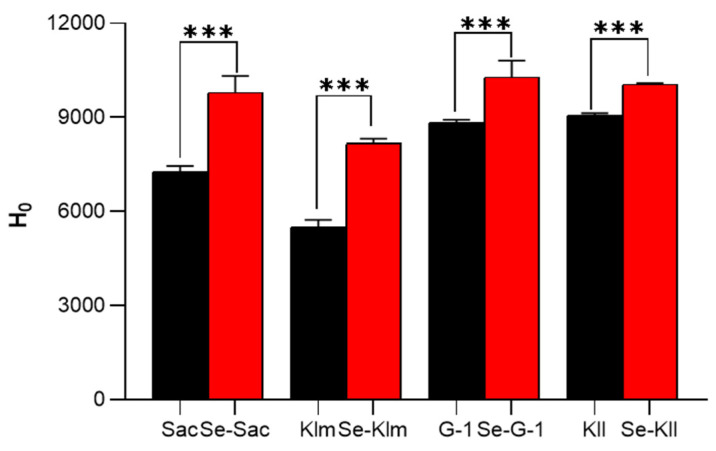
Surface hydrophobicity (H_0_) of protein extracts derived from yeast strains, cultivated with and without selenium supplementation. Note: *** indicates *p* < 0.001. Sac = *Saccharomyces cerevisiae*; Klm = *Kluyveromyces marxianus*; G-1 = *Torulaspora delbrueckii*; Kll = *Kluyveromyces lactis*.

The changes in surface hydrophobicity of four yeast proteins following Se enrichment are illustrated in Figure 4. Compared to the control group, the surface hydrophobicity of all selenium-enriched yeast proteins exhibited a significant increase. Notably, the percentage increases for Se-Sac (34.6%) and Se-Klm (48.7%) were greater than those observed for Se-Kll (10.8%) and Se-G-1 (16.3%). Hydrophobic amino acids, such as Trp, Tyr, Met, Cys, Phe, and Pro, are typically sequestered within the interior of the protein structure. The binding of Se to the protein results in the exposure of these hydrophobic amino acids to the external environment, thereby influencing the protein’s structural integrity and correlating with its antioxidant activity [65].

### 3.4. Enhanced Antioxidant Capacity of Selenoproteins

**Figure 5:**
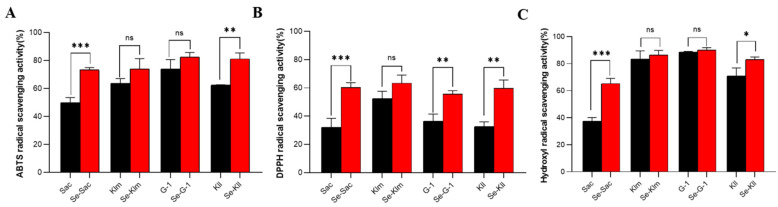
Antioxidant capacity of protein extracts derived from yeast strains cultivated with and without selenium supplementation: (**A**) ABTS radical scavenging rate; (**B**) DPPH radical scavenging rate; (**C**) Hydroxyl radical scavenging rate. Sac = *Saccharomyces cerevisiae*; Klm = *Kluyveromyces marxianus*; G-1 = *Torulaspora delbrueckii*; Kll = *Kluyveromyces lactis*. Note: * indicates *p* < 0.05; ** indicates *p* < 0.01; *** indicates *p* < 0.001; ns indicates no significance.

This study evaluated the antioxidant capacity of selenium-enriched yeast proteins before and after enrichment using DPPH^•^, ABTS^•+^ and ^•^OH assays. Compared to the control group, all selenium-enriched proteins exhibited enhanced scavenging rates for the three types of free radicals (Figure 5). Notably, the free radical scavenging activities of DPPH^•^, ABTS^•+^, and ^•^OH in Se-Sac and Se-Kll were significantly higher than those in Sac and Kll, respectively. In contrast, the free radical scavenging activities of proteins derived from the other two types of yeast did not show significant changes after Se enrichment, except for the DPPH^•^ scavenging activity of the protein derived from Se-G-1, which was significantly higher than that from G-1.

Oxidation is a significant contributor to various diseases, including cardiovascular disorders, cancer, Alzheimer’s disease, and aging [66]. Therefore, preventing free radical-induced oxidative damage is crucial, and natural, safe antioxidants are highly valued. Selenium-enriched yeast protein not only serves as an effective dietary source of Se but also exhibits remarkable antioxidant activity. Our findings demonstrate that the antioxidant capacity of *S*. *cerevisiae*-derived protein (Sac) is substantially enhanced following Se enrichment. This enhancement can be partially attributed to its high selenium-binding affinity and the presence of weaker Se–X bonds, which facilitate electron transfer to free radicals [52]. More importantly, the structural reorganization induced by Se incorporation plays a decisive role in enhancing antioxidant function.

**Figure 6:**
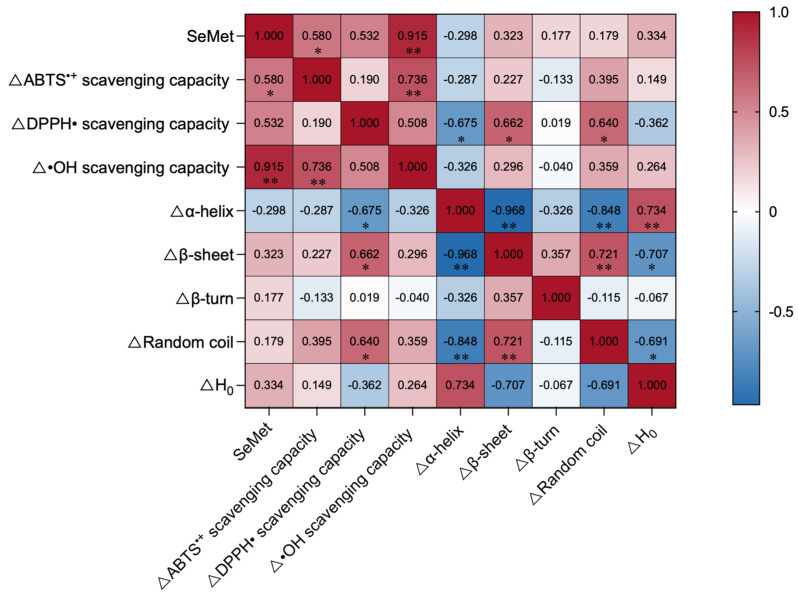
Pearson correlation heatmap illustrating the relationships between the percentage changes in protein secondary structural elements and the percentage changes in antioxidant capacities following selenium enrichment. The color scale represents the correlation coefficient (r), with red indicating positive correlations and blue indicating negative correlations. * denote statistical significance (*p* < 0.05); ** denote statistical significance (*p* < 0.01).

As summarized in Table 3 and illustrated in Figure 4, Se enrichment universally induced a notable reduction in the rigid β-sheet content across all yeast strains. Specifically, Se-Sac exhibited a clear decrease in β-sheet alongside an increase in random coil and H_0_. This indicates a transition from a rigid core toward a looser, more flexible, and solvent-exposed protein architecture, likely due to the incorporation of bulky selenium atoms disrupting native hydrogen bond networks. A Pearson correlation analysis was performed based on incremental changes (Figure 6). Notably, the changes such as Δβ-sheet (r = 0.662, *p* < 0.05) and Δrandom coiling (r = 0.640, *p* < 0.05) show a positive correlation with the ΔDPPH^•^ effect. Moreover, the increment in α-helix content demonstrated a significant negative correlation with the enhancement of ΔDPPH^•^ (r = −0.675, *p* < 0.05). In addition, there is a very strong and significant positive correlation between the absolute proportion of SeMet and the improvement of hydroxyl radical scavenging ability (r = 0.915, *p* < 0.01), and a significant positive correlation with the change in ABTS scavenging ability (r = 0.580, *p* < 0.05). Collectively, the targeted substitution of SeMet directly induces an increase in disordered structures. This SeMet-driven structural relaxation fundamentally enhances the spatial accessibility and contact area between internal active sites and free radicals. Collectively, the inherent high reactivity of SeMet combined with this conformational change leads to a profound enhancement in overall antioxidant efficacy, resulting in superior scavenging capabilities against DPPH^•^, ABTS^•+^, and ^•^OH. Furthermore, the elevated surface hydrophobicity facilitates the localization of antioxidants at lipid-aqueous interfaces where oxidation occurs [67]. Therefore, *S*. *cerevisiae* can convert the absorbed selenium into selenomethionine at a rate of 85.80%, and it shows a decrease in β-sheet, an increase in random coil, and enhanced hydrophobicity. It effectively utilizes this synergistic mechanism to achieve the most significant improvement in antioxidant performance.

**Figure 7:**
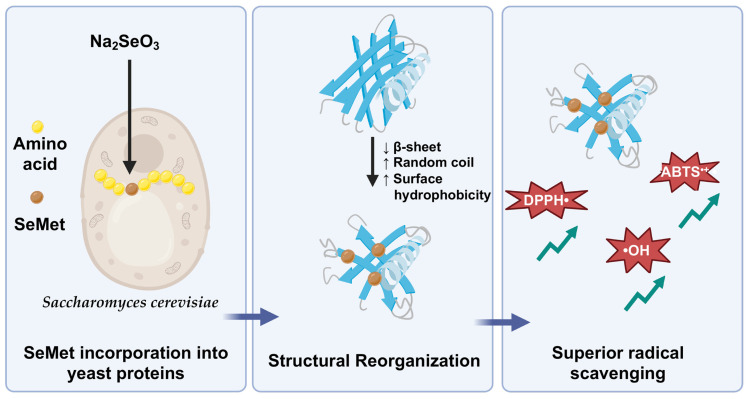
Schematic representation of the structure–function paradigm of selenium-enriched yeast proteins. The downward (↓) and upward (↑) arrows denote a decrease and an increase in the corresponding parameters, respectively. Created in BioRender. Ma, Y. (2026) https://BioRender.com/cw41ezc (accessed on 6 March 2026).

In contrast, despite achieving high total selenium accumulation, strains like Kll exhibited a relatively low SeMet conversion rate. Consequently, even with an increase in disordered structures, their antioxidant enhancement remained markedly inferior to that of Se-Sac. This clearly demonstrates that functional improvement is not merely a byproduct of gross selenium content, but is intrinsically driven by the targeted bioconversion of SeMet and the associated structural relaxation. This finding highlights the distinct advantage of our structure–function approach over conventional practices: while routine screening often prioritizes strains with the highest total selenium yield (e.g., Kll), our assessment proves that functional superiority is synergistically governed by specific selenium speciation and favorable conformational unmasking (as seen in Sac). Therefore, the precise chemical form of selenium and its structural impact, rather than simple elemental addition, are the true determinants of antioxidant efficacy in selenium-enriched proteins (Figure 7).

### 3.5. Technical Challenges and Future Directions for Commercial Applications

Our findings not only elucidate the fundamental mechanisms but also provide a scientific rationale for the targeted selection and design of selenium-enriched yeast. The established interrelationship among Se speciation, protein structure, and antioxidant function represents a shift from the conventional paradigm of merely maximizing total Se content toward a more sophisticated strategy of precision design. For instance, Se-Sac, characterized by its high SeMet content and significantly enhanced antioxidant capacity, emerges as an ideal candidate for the development of antioxidant nutraceuticals aimed at mitigating chronic diseases associated with oxidative stress [59]. Currently, selenium-enriched yeast proteins are utilized extensively globally as dietary supplements, food additives, and functional food ingredients due to their high bioavailability compared to inorganic Se sources. While modern production methods utilizing common species can achieve remarkable total Se contents—up to 3.89 mg/g dry weight with SeMet constituting the vast majority of selenoamino acids [68]—our study provides a critical advancement for quality control. We propose that industrial screening should no longer rely exclusively on total Se accumulation. Instead, identifying strains capable of beneficial structural reorganization should become a new practical criterion for optimizing antioxidant efficacy.

Despite these promising prospects, several technical challenges must be addressed for successful commercialization. First, maximizing biotransformation efficiency while maintaining yeast viability remains a critical bottleneck. The observed growth inhibition of yeast strains in this study (10.4–27.7%) indicates that elevated Se concentrations are inherently toxic to the yeast itself, which limits the ultimate biomass yield in large-scale industrial bioreactors. Second, scaling up the fermentation process introduces complexities in maintaining structural consistency. Ensuring that the beneficial SeMet-driven structural relaxation (i.e., the transition toward a looser, more solvent-exposed conformation) consistently occurs across massive industrial batches requires meticulous control of fermentation parameters. Furthermore, standardizing the extraction of total soluble intracellular proteins from complex yeast matrices to ensure batch-to-batch functional reproducibility poses a significant engineering challenge.

Although our comparative screening at a standardized concentration successfully elucidated the relative structure–function relationships, it does not provide absolute pharmacological kinetics. Future investigations must prioritize the determination of comprehensive dose–response curves and IC_50_ values for these specific selenoproteins to guide precise dosage formulations for clinical or dietary applications. Additionally, while the organic forms of Se (particularly SeMet) demonstrate significantly lower toxicity compared to inorganic species—providing crucial evidence for their safety as dietary sources—our study was primarily conducted in vitro. Moving forward, research must transition to in vivo animal models and clinical trials to validate the metabolic absorption, bioavailability, and specific health benefits (such as immune regulation, neuroprotection, and hyperglycemic control) of these structurally optimized proteins within complex biological systems [69,70,71].

## 4. Conclusions

This study establishes a critical structure–function paradigm for selenium-enriched yeast proteins, demonstrating that the incorporation of selenomethionine and the associated structural reorganization, rather than gross Se accumulation alone, is the primary driver of enhanced antioxidant functionality. Our findings challenge conventional purely quantitative evaluation methods by revealing that specific conformational shifts, such as the profound SeMet-driven structural relaxation observed in *S*. *cerevisiae*, are essential for maximizing free radical scavenging capabilities. This underscores the fundamental necessity of evaluating protein structural dynamics alongside elemental biotransformation. Moving forward, future research should transition from in vitro chemical assays to in vivo animal models and clinical trials. This is crucial to validate the metabolic absorption, bioavailability, and specific health benefits of these structurally optimized selenoproteins within complex biological systems. From an industrial perspective, these insights offer a novel and precise criterion for strain selection and process optimization. By prioritizing yeast strains that couple high biotransformation efficiency with beneficial structural remodeling, manufacturers can transcend traditional selection metrics. This structure–function paradigm provides a robust scientific foundation for the targeted design, quality control, and scalable production of the next generation of high-potency, selenium-enriched functional foods and nutraceuticals.

## Figures and Tables

**Table 1:** Biomass (dry cell weight), total selenium, protein yield, and protein-bound selenium content in yeast with and without selenium supplementation.

Yeast	Biomass (g/L)	Se Content in Biomass (μg/g d.w.)	Protein Yield (mg/g Biomass)	Se Content in Protein (μg/g)
Sac	5.00 ± 0.03	2.99 ± 0.46	535.95 ± 9.37	5.49 ± 2.20
Se-Sac	4.48 ± 0.23 *	1915.68 ± 107.00 ***	484.00 ± 12.44 **	3529.55 ± 132.07 ***
Klm	5.09 ± 0.09	2.05 ± 0.18	499.35 ± 4.09	5.02 ± 1.13
Se-Klm	3.68 ± 0.14 ***	1389.12 ± 237.16 ***	521.79 ± 10.63 *	2781.03 ± 275.80 ***
G-1	0.52 ± 0.02	1.67 ± 0.23	585.55 ± 9.37	3.29 ± 1.29
Se-G-1	0.48 ± 0.02 ^ns^	1164.00 ± 297.27 ***	538.32 ± 8.91 **	1973.79 ± 159.82 ***
Kll	4.94 ± 0.11	3.88 ± 0.27	594.99 ± 17.47	7.04 ± 0.27
Se-Kll	4.85 ± 0.02 ^ns^	2858.70 ± 245.22 ***	479.28 ± 9.37 ***	4884.13 ± 405.66 ***

Note: Sac = *Saccharomyces cerevisiae*; Klm = *Kluyveromyces marxianus*; G-1 = *Torulaspora delbrueckii*; Kll = *Kluyveromyces lactis*. Data were analyzed using a paired *t*-test for within-strain comparisons (e.g., Se-Sac vs. Sac), with significance indicated by asterisks (* *p* < 0.05, ** *p* < 0.01, *** *p* < 0.001, ns, not significant).

**Table 2:** Absolute concentrations and relative percentage distributions of extractable selenium species in selenium-enriched yeast proteins.

Protein	Parameter	SeCys_2_	MeSeCys	Se(IV)	SeMet	Se(VI)
Se-Sac	Conc. (μg/g)	0.81 ± 0.32 ^c^	1.94 ± 0.58 ^a^	0.45 ± 0.19 ^a^	19.60 ± 0.96 ^a^	0.06 ± 0.02 ^a^
	Rel. (%)	3.57 ± 1.56 ^C^	8.42 ± 2.34 ^C^	1.93 ± 0.77 ^BC^	85.80 ± 1.48 ^A^	0.28 ± 0.09 ^C^
Se-Klm	Conc. (μg/g)	2.45 ± 0.44 ^b^	1.38 ± 0.21 ^a^	0.32 ± 0.03 ^a^	0.12 ± 0.01 ^d^	0.03 ± 0.01 ^b^
	Rel. (%)	56.71 ± 7.55 ^A^	32.26 ± 6.46 ^A^	7.56 ± 1.07 ^A^	2.74 ± 0.17 ^D^	0.73 ± 0.19 ^B^
Se-G-1	Conc. (μg/g)	7.03 ± 0.88 ^a^	1.82 ± 0.24 ^a^	0.38 ± 0.10 ^a^	2.15 ± 0.32 ^c^	0.02 ± 0.01 ^b^
	Rel. (%)	61.56 ± 4.41 ^A^	15.94 ± 1.30 ^B^	3.29 ± 0.68 ^B^	19.01 ± 3.89 ^C^	0.21 ± 0.06 ^C^
Se-Kll	Conc. (μg/g)	1.97 ± 0.29 ^b^	0.39 ± 0.03 ^b^	0.05 ± 0.01 ^b^	3.81 ± 0.18 ^b^	0.08 ± 0.01 ^a^
	Rel. (%)	31.16 ± 2.46 ^B^	6.24 ± 0.96 ^C^	0.86 ± 0.05 ^C^	60.53 ± 1.36 ^B^	1.21 ± 0.19 ^A^

Note: Sac = *Saccharomyces cerevisiae*; Klm = *Kluyveromyces marxianus*; G-1 = *Torulaspora delbrueckii*; Kll = *Kluyveromyces lactis*. Different lowercase letters (a–d) in the same column indicate significant differences in absolute concentrations (Conc.) among different yeast strains at *p* < 0.05. Different uppercase letters (A–D) in the same column indicate significant differences in relative percentages (Rel.) among different yeast strains at *p* < 0.05.

**Table 3:** Analysis of secondary structure in protein extracts derived from yeast strains cultivated with and without selenium supplementation by CD spectroscopy.

Protein	Percentage Composition of Secondary Structure (%)			
	α-Helix	β-Sheet	β-Turn	Random Coil
Sac	1.90 ± 0.26	64.37 ± 1.23	0.23 ± 0.40	33.47 ± 0.61
Se-Sac	4.63 ± 0.84 **	55.27 ± 3.33 *	2.70 ± 1.15 *	37.43 ± 1.43 *
Klm	2.23 ± 0.06	61.17 ± 0.49	0.00 ± 0.00	36.63 ± 0.40
Se-Klm	29.77 ± 0.35 ***	36.90 ± 0.90 ***	0.00 ± 0.00 ^ns^	33.40 ± 0.56 ***
G-1	0.00 ± 0.00	57.80 ± 0.71	1.65 ± 0.07	40.50 ± 0.85
Se-G-1	0.00 ± 0.00 ^ns^	50.50 ± 1.98 ***	6.70 ± 0.28 ***	42.80 ± 1.70 *
Kll	21.35 ± 0.07	51.80 ± 1.13	0.00 ± 0.00	26.85 ± 1.06
Se-Kll	14.70 ± 0.28 ***	45.95 ± 0.21 ***	0.00 ± 0.00 ^ns^	39.35 ± 0.49 ***

Note: Sac = *Saccharomyces cerevisiae*; Klm = *Kluyveromyces marxianus*; G-1 = *Torulaspora delbrueckii*; Kll = *Kluyveromyces lactis*. Data are expressed as the mean ± standard deviation (SD). *, **, *** denote significant differences between selenium-enriched and control proteins within each strain (*p* < 0.05, 0.01, 0.001); ns indicates not significant.

## Data Availability

The original contributions presented in the study are included in the article/Supplementary Materials. Further inquiries can be directed to the corresponding author.

## Supplementary Materials

The following supporting information can be downloaded at: https://www.mdpi.com/article/10.3390/antiox15030370/s1. Figure S1. Representative raw circular dichroism (CD) spectra of protein extracts derived from yeast strains cultivated with and without selenium supplementation. Sac = *Saccharomyces cerevisiae*; Klm = *Kluyveromyces marxianus*; G-1 = *Torulaspora delbrueckii*; Kll = *Kluyveromyces lactis*.
