# Supplementary material for: Elucidating the Effects of Selenium Enrichment on the Structure and Antioxidant Properties of Selenium-Containing Proteins in Yeast Cells
Source: Antioxidants (Basel). 2026 Mar 15;15(3):370. doi: 10.3390/antiox15030370 (PMC13024480; doi:10.3390/antiox15030370)
Supplement: Supplementary file 1 [file antioxidants-15-00370-s001.zip › antioxidants-4156723-supplementary.pdf]

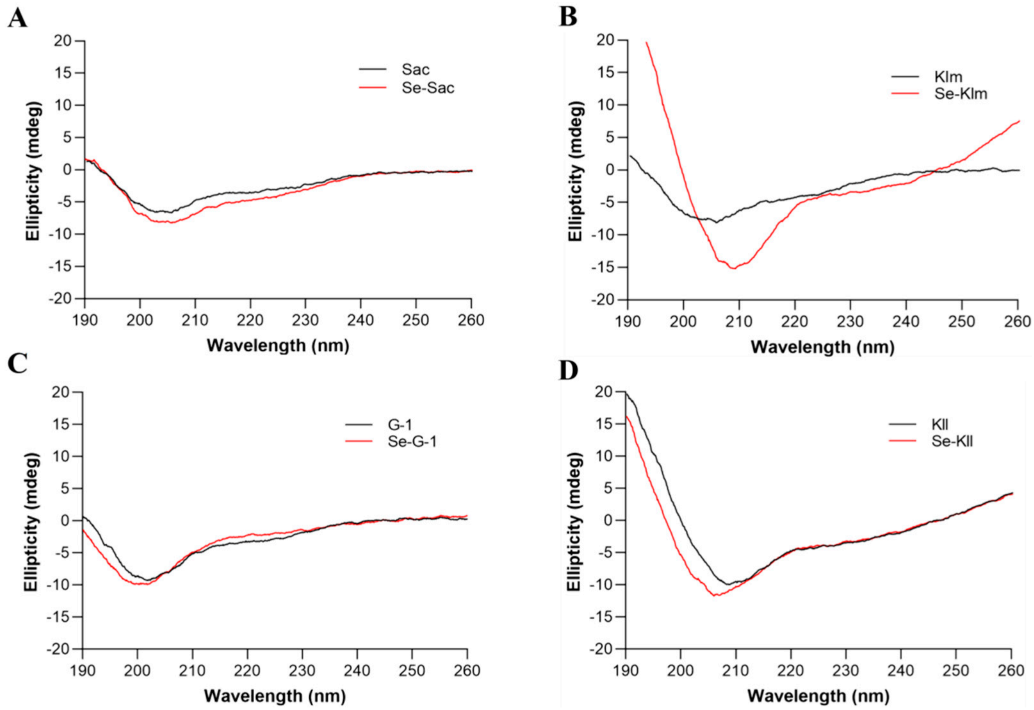

Figure S1 Representative raw circular dichroism (CD) spectra of protein extracts derived from yeast strains cultivated with and without selenium supplementation. (**A**) Sac, (**B**) Klm, (**C**) G-1, (**D**) Kll. Sac = *Saccharomyces cerevisiae*; Klm = *Kluyveromyces marxianus*; G-1 = *Torulaspora delbrueckii*; Kll = *Kluyveromyces lactis*.
